# Construction and validity of an educational video to prevent immunization errors

**DOI:** 10.1590/0034-7167-2023-0010

**Published:** 2023-10-09

**Authors:** Naiara Cristina Silva Simões, Laís Oliveira de Moraes Tavares, Carlos Miguel Bolognani da Silva, Samuel Barroso Rodrigues, Stênio Henrique Oliveira, Eliete Albano de Azevedo Guimarães, Valeria Conceição de Oliveira

**Affiliations:** I Universidade Federal de São João del-Rei. Divinópolis, Minas Gerais, Brazil; II Universidade de Itaúna. Itaúna, Minas Gerais, Brazil

**Keywords:** Vaccination, Medical Errors, Instructional Film and Video, Educational Technology, Nursing., Vacunación, Errores de Medicación, Película y Vídeo Educativos, Tecnología Educacional, Enfermería., Vacinação, Erros de Medicação, Filme e Vídeo Educativo, Tecnologia Educacional, Enfermagem.

## Abstract

**Objective:**

to construct and validate an orientation video, based on a low-fidelity clinical simulation scenario, to prevent immunization errors.

**Methods:**

a methodological study with video construction, validated in two stages by different audiences. Content was selected based on a realistic simulation scenario of the vaccine administration process to a patient-actor. Items with concordance greater than 0.8 and 0.6 were considered valid, verified using the Content Validity Index (CVI) and the Content Validity Ratio (CVR), respectively.

**Results:**

judges’ CVI had an average of 97.5%, and CVR, 0.9, and health professionals’ CVI, 95.4%, and CVR, 0.8. Successes in administering vaccines were addressed, such as careful reading of labels, double-checking the vaccine, distractions/interruptions and error reporting.

**Conclusions:**

the video was constructed and validated in terms of content, and can be used in training professionals working in vaccination.

## RESUMO

**Objetivo:**

construir e validar um vídeo de orientação, baseado em um cenário de simulação clínica de baixa fidelidade, para prevenção de erros de imunização.

**Métodos:**

estudo metodológico com construção de vídeo, validado em duas etapas por público distinto. Selecionou-se o conteúdo por um cenário de simulação realística do processo de administração de vacina a um paciente-ator. Consideraram-se válidos itens em concordância superior a 0,8 e 0,6, verificados por meio do Índice de Validação de Conteúdo (IVC) e do *Content Validity Ratio* (CVR), respectivamente.

**Resultados:**

o IVC dos juízes teve média de 97,5%, e CVR, de 0,9, e IVC dos profissionais de saúde, de 95,4%, e CVR, de 0,8. Abordaram-se os acertos na administração das vacinas, como leitura atenta dos rótulos, dupla checagem da vacina, distrações/interrupções e notificação do erro.

**Conclusões:**

o vídeo foi construído e validado quanto ao conteúdo, podendo ser utilizado na capacitação de profissionais atuantes em vacinação.

## RESUMEN

**Objetivo:**

construir y validar un video de orientación, basado en un escenario de simulación clínica de baja fidelidad, para prevenir errores de inmunización.

**Métodos:**

estudio metodológico con construcción de video, validado en dos etapas por diferentes audiencias. El contenido fue seleccionado en base a un escenario de simulación realista del proceso de administración de vacunas a un paciente-actor. Se consideraron válidos los ítems con concordancia superior a 0,8 y 0,6, verificados mediante el Índice de Validación de Contenido (IVC) y el Índice de Validez de Contenido (CVR), respectivamente.

**Resultados:**

el CVI de los jueces tuvo una media de 97,5% y CVR de 0,9 y el de los profesionales de la salud de 95,4% y CVR de 0,8. Se abordaron los éxitos en la administración de vacunas, como la lectura cuidadosa de las etiquetas, la verificación doble de la vacuna, las distracciones/interrupciones y la notificación de errores.

**Conclusiones:**

el video fue construido y validado en cuanto al contenido, pudiendo ser utilizado en la formación de profesionales que actúan en vacunación.

## INTRODUCTION

Vaccination is a strategy capable of impacting the control or elimination of vaccine-preventable diseases^(1-2)^. However, safe vaccination is a worldwide concern and a determining factor for the success or failure of immunization programs^(3-4)^. In recent decades, there has been a significant increase in the number of vaccines applied to children and adults and, consequently, the increase in the occurrence of immunization errors globally^(5-6)^.

Immunization error (IE) is defined as any preventable event arising from the inappropriate use of immunobiological agents that may generate negative impacts, such as impaired immunization of a person, Events Supposedly Attributable to Vaccination or Immunization (ESAVI), in addition to increased costs for the health service and reduced confidence in Brazilian National Immunization Programs (PNI - *Programas Nacionais de Imunizações*)^(5,7-9)^.

ESAVI reporting resulting from IE have been increasing, which highlights a worrying current scenario. Approximately one third of patients have at least one experience with some type of IE. National and international literatures demonstrate that IE are associated with errors in vaccine preparation, incorrect route of administration, incorrect dose, incorrect interval and errors in storage^(6-7,9-13)^.

A systematic review analyzed the prevalence of IE documented between 2009 and 2018, and found that, for every 10,000 doses applied, 1.15 were administered erroneously^(14)^. In Brazil, vaccination is an action carried out by the nursing team, more often by nursing technicians, under the supervision of nurses, considering that nurses have other managerial and care duties^(15)^. It is important to guarantee frequent updates on care practices, thinking about the adoption of measures that help professionals not to make mistakes, guaranteeing safe care. Permanent health education is a learning process in the service, where learning and teaching are associated with the daily life of this scenario. It must be planned based on the questioning of everyday life so that it is effective and generates reflection among professionals and, consequently, actions for improvements and learning^(9,15-16)^.

In this sense, the use of technologies has proven to be a promising and important tool both for student and professional learning^(17-18)^. The construction of an educational video is presented as a didactic and technological instrument that provides knowledge and makes the educator-learner interaction more effective. The educational video approaches the everyday environment, in addition to being an attractive and contextualizing medium, motivating learning^(19-20)^.

Considering the above, the development of an educational video for IE prevention could contribute to training workers and be disseminated, aiming at dissemination of knowledge, making it more accessible to nursing professionals who work in the vaccine room.

The research aimed to build and validate an orientation video based on a clinical simulation scenario for IE prevention.

## OBJECTIVE

To construct and validate an orientation video, based on a low-fidelity clinical simulation scenario, for IE prevention.

## METHODS

### Ethical aspects

The study was submitted and approved by the Research Ethics Committee of the *Universidade Federal de São João del-Rei* - Center-West *Campus*. The signature of the Term of Authorization for the Use of Image and Voice was requested, for the video participants, and the Informed Consent Form, for judges and target audience, in addition to the Term of Consent, for the guardian of the child who participated from the video scenes.

### Study design, place and period

This is a methodological study, carried out from December 2020 to November 2021, in a municipality in Minas Gerais, for the preparation and validity of a video with guidelines to prevent IE. For the video construction and development, the phases of pre-production, production and post-production followed^(21-22)^.

### Population or sample; inclusion and exclusion criteria

There is no consensus in the literature regarding the number of judges needed to obtain validity^(23)^. The video went through two stages of validity: one by judges and another by the target audience.

For video validity, 27 judges were identified to participate in the study, however 13 responded to the invitation sent by email. Participants were identified through a search on the *Plataforma Lattes* and selection of professors from different universities, with the requirement of practical or academic experience of at least five years in the research topic.

For the video validity by the target audience, nursing professionals who worked in vaccination with at least one year of experience and who agreed to participate in the research were invited. Professionals were selected who met the inclusion criteria established by the researchers and who had an average of 30 minutes available to watch the video and respond to the assessment questionnaire. Validity by the target audience consisted of 3 nurses and 9 nursing technicians.

### Study protocol

The pre-production phase was based on three stages: construction of a clinical simulation scenario; video script/storyboard elaboration; and validity of this by judges.

Initially, a clinical simulation scenario was built, presenting the vaccine administration process by a nursing professional with extensive experience in vaccinating a patient-actor. A total of 5 researchers, 4 graduate students and a nurse participated in simulation. Among them are the supervisor of this research, a specialist in the area of immunization as well as a facilitator with experience in the area of realistic simulation.

For each event in simulation, specific actions were developed that professionals are expected to carry out during vaccination, such as assessing the vaccination situation, screening the vaccinated, registering in the information system, administering the vaccine, among others. All steps of realistic simulation were recorded. As for fidelity, it is perceived that simulation achieves an approximation with the daily life experienced by professionals, making it useful for identifying problems and solving them. The use of realistic simulation brings significant impacts on the structuring of teaching and learning, allowing the link between theory and practice, contributing to the meaningful learning of learning by doing^(24)^.

After the realistic simulation, a debriefing was carried out, with the objective of identifying safe practices in IE prevention. Debriefing is an important moment for participants to reflect on what was covered in simulation, identifying the positive and negative points and what could have been done differently^(17)^.^.^

The educational video’s script was built based on safe vaccination practices identified in realistic simulation and debriefing, on the norms of procedures in the Brazilian PNI’s vaccination room and on the experience of the advisor of this project, a researcher in the field of vaccination. The script should tell the film crew or readers what should happen in the video by describing the scenes^(25)^.

In order to guide the video production, the script was divided into three columns: thematic, which indicates what is being addressed; narration and audio, which describes what will be said by the narrator during the video; and scenes and images, which appear throughout the video, related to the theme and narration.

After preparing the script, the storyboard was created with the same content as the script, following the same division. The storyboard detailed the scenes, photos/illustrations and testimonials used. In this way, the judges could preview the video and perform the assessments.

After preparing the video script/storyboard content, the material was sent to the 13 judges who agreed to participate in the validity study.

For the script/storyboard analysis with the committee of judges, a questionnaire validated in a previous study and adapted to the IE theme was used^(22)^. The instrument consisted of five chunks of statements, considered as evaluative items of the script: objectives (purposes, goals or ends of the video); content (video presentation, form and structure); relevance (characteristics of images and scenes proposed in the script); environment (scenario assessment); and verbal language (language used in the video). A five-point Likert scale was used, where judges should choose the option that corresponded to their opinion: Strongly Agree (SA); Agree (S); Neither agree/disagree (NN); Disagree (D); and Strongly Disagree (SD). A space was allocated for the personal interpretative analysis of a committee of judges.

The next stage, video production, consisted of filming the scenes present in the validated script. The images and audio were recorded by the researchers, who had the help of a professional trained in journalism regarding the best angle to record, suitable lighting, correct positioning of the cameras and ways to improve the audio.

Volunteer nursing professionals who work in vaccination rooms participated in the recording of scenes and testimonies. The choice of professionals was guided by their familiarity with the topic of vaccination and proximity to the researchers. After agreeing to participate in the recordings, professionals signed the Term of Authorization for the Use of Image and Voice. The script texts were sent to the professionals to be rehearsed prior to the recordings.

The recordings were made in two vaccination rooms, where nursing professionals who participated in the video worked, and some scenes from the realistic simulation scenario were used in the video. The decision to film in the vaccination rooms was due to the need to reproduce reliable scenes that portrayed reality and also a concrete physical environment. And the use of two rooms was to facilitate the participation of volunteer professionals. Images of the rooms, simulation of vaccination practice, testimonials from professionals, photos and audiovisual effects were used.

For the video presentation and narration, there was the participation of a professor who teaches vaccination and has experience in performing arts. In this phase, the presence of the researchers was essential to assess whether the video’s objective was being achieved^(21)^.

In the post-production phase, the video editing, finalization and final organization was carried out by a contracted company, specialist in video editing, and all editing and montage was done together with the researchers so that the video followed the validated script. Windows MovieMaker, version 16.4.3528.0331, FormatFactory 1.70 and PhotoScape, version 3.6.2 were used. Using this software, scenes were selected, edited and organized, the soundtrack was chosen and edited and visual effects were inserted, images were assembled and paired, characters, subtitles and figures were superimposed.

After completing the post-production phase, the video was assessed by the target audience, consisting of 3 nurses and 9 invited nursing technicians. The professionals were approached at the health units to watch and assess the video on a date previously scheduled with the researcher. In the health units where each professional worked, the video was shown, and each one was asked to assess whether the language and images were clear and easy to understand or whether there was any suggestion. For assessment, the same instrument was used^(22)^, applied to expert judges, also organized on a Likert-type scale with five response levels.

### Analysis of results, and statistics

Item suitability analysis was performed using the Content Validity Index (CVI). The calculation of this index was carried out by dividing the sum of responses considered suitable (SA and A) by the total number of responses. Items that obtained concordance rates greater than or equal to 80% (0.8) among judges and the target audience were considered valid.

The Content Validity Ratio (CVR - Content validity Ratio). While CVI measures the proportion of judges and professionals with answers (SA+A), the CVR compares this proportion with the expected number, if judges/professionals were responding at random [(CVR = n _e_ - (N/2) / (N/2)], where “n_e_” is the number of judges and professionals who classified each item as “SA or A” and “N” is the total number of judges and professionals responding. The minimum CVR expected for this study was of 0.6 for judges and 0.5 for the target audience^(26)^. After the assessments, suggestions were accepted and the final version of the video was defined based on suggestions made by judges and the target audience.

## RESULTS

Realistic simulation took place in a vaccination room where a nurse worked with the simulated vaccinated according to the previously established objective and the others were instructed to observe the scene, with this stage lasting 30 minutes. After the realistic simulation, the debriefing was carried out, which constituted a moment of joint discussion coordinated by the facilitator and the researcher and this stage lasted about 60 minutes.

During debriefing, it was discussed that the procedures for preventing IE begin before administering the vaccine, making it necessary to check the person’s age, the vaccine to be administered, in addition to vaccine screening. Another fundamental point is to check the physical vaccine card and the vaccination records contained in the immunization information system and register the immunobiological that will be administered.

During administration, it is of fundamental importance to read the vials carefully, as many immunobiological agents have similar vials and can be easily confused. Presenting the vaccine vial to the person vaccinated or responsible is a form of double checking. Pay attention to how the immunobiological is presented, whether it should be reconstituted or not. The route of administration, the correct dosage, needle choice and the correct place for immunobiological agent administration. Moreover, the importance of the vaccination room structure and organization and the importance of maintaining the immunobiological cold chain were also addressed.

Another theme addressed in debriefing was the possible occurrence of interruptions during the immunization process and the possible ways of preventing them. The need to know the steps that must be followed in cases of error occurrence was pointed out. Based on the debriefing discussion, a script with 18 scenes was prepared and sent to validity.

There were two moments of validity, validity by judges and by the target audience. The expert committee was composed of 13 judges, 09 (69.2%) female, aged between 33 and 60 years. Most participants are graduates in nursing (92.3%), and most of them (46.3%) have a postdoctoral degree.

Table 1 shows the results of CVI and CVR on judges’ validity for items assessed in the categories: objectives, content, relevance, environment and verbal language.

**Table 1:** Consensus obtained on validity by judges in each assessed item of the script, Divinópolis, Minas Gerais, Brazil, 2021

Categories	CVI (%)	CVR
1. Objectives		
1.1. Objectives are consistent with the objectives proposed in the research	100	1.0
1.2. Objectives are consistent with the Brazilian National Immunization Program recommendations	100	1.0
1.3. Objectives are consistent with practice in the vaccination room	100	1.0
1.4. Objectives are suitable to be carried out	99	0.8
2. Content		
2.1. Content presented in the script corresponds to the objectives proposed in the work	100	1.0
2.2. Content facilitates the teaching-learning process on the subject	100	1.0
2.3. Content allows understanding the theme	100	1.0
2.4. Content follows a logical sequence	100	1.0
2.5. Content incorporates the necessary information to promote immunization error prevention	80	0.7
2.6. Content has all the material resources necessary to promote immunization error preventions	80	0.7
2.7. Information in the script is correct	100	1.0
3. Relevance		
3.1. Images and scenes described illustrate important aspects for promoting immunization error prevention	100	1.0
3.2. Images and scenes are relevant for nursing workers to adopt behaviors that promote error immunization prevention	100	1.0
3.3. Images and scenes allow the transfer and generalization of learned content to different contexts of the work environment	80	0.7
4. Environment		
4.1. Setting is suitable for video production	100	1.0
4.2. Setting is suitable for learning the theme	100	1.0
5. Verbal Language		
5.1. Verbal language in the script is suitable for the target audience	100	1.0
5.2. Verbal language is easy to assimilate	100	1.0

Note: CVI - Content Validity Index; CVR - Content Validity Ratio.

There was unanimous agreement (100%) of judges regarding the following categories: environment and verbal language. In the objective category, item 1.4 showed 99% agreement. One of the judges justified that the video script covered much more than what was proposed in the research objectives, justifying that the video will contribute to the error reporting culture, increasing the study relevance.

In the content category, in items 2.5 and 2.6, it was suggested to add a speech about the consequence of the error for professionals, such as negative feelings, which becomes the event’s second victim. Another suggestion was the modification of “*Zé Gotinha*”’s expression (PNI mascot), used to illustrate a scene in the script. A judge suggested replacing “*Zé Gotinha*’s negative expression”, which suggests disapproval by IE, which is a form of moral punishment, with an expression of sadness, compassion on the part of patients for the damage that has occurred. Another judge recommended that, instead of just talking about the importance of placing a sign with the words, “In attendance, wait”, in the vaccination room, it would be interesting to film a professional doing this, encouraging this to be an action in every vaccination service.

In the relevance category, in item 3.3, a judge pointed out that the image of a child smiling, at the end of the video, could bring ambiguous interpretations to the vaccination process, that of the good of vaccination, for disease prevention, and that of the non-’real’ imaginary, since, normally, children cry when they are vaccinated.

Reformulations of excerpts from the narration were suggested, with a view to making them more understandable by professionals. It was suggested to change the title of the video from “Immunization error: forms of prevention” to “Preventive strategies for immunization errors”. It was recommended to include information on the organization of immunobiological agents in the refrigerated chamber and their identification with labels, aiming at safety in the vaccination room. The suggested changes were accepted, and the necessary changes were made. Judges’ total CVI and CVR were 97.5% and 0.9, respectively.

Video validity by target audience was carried out by 12 professionals who work in immunization rooms, 3 nurses (25%) and 9 nursing technicians (75%). All participants had worked in the immunization room for more than one year.

In professionals’ assessment, the CVR and the total CVI for the categories were: objectives (96% and 0.7); content (95% and 0.6); relevance (95% and 0.8); environment (91% and 0.8); and verbal language (100% and 1.0). The video was considered relevant, with suitable and understandable language, reflecting the vaccination room’s reality. Two professionals reported that the sound could have been a little louder. Due to the fact that the recording took place during the COVID-19 pandemic, some nursing professionals wore a mask, which may have impaired the sound.

The final version of educational video “Preventive strategies for immunization errors” ended with 12 minutes and 35 seconds.

## DISCUSSION

The simulation scenario was important to represent a safe vaccination and direct the debriefing to the discussion on IE prevention. Realistic simulation brings significant impacts on the teaching and learning structure, for being characterized as a strategy that replicates precisely an event, situation, environment or scenario, allowing the link between theory and practice, contributing to the meaningful learning of learning by doing^(24)^.

The methodological route, used in the educational video construction and validity, was fundamental for the development of a quality material. The script/storyboard construction, based on realistic simulation and debriefing as well as the use of scientific literature and the wide experience of professionals in the subject can justify the almost totality of judges’ agreement.

Educational technology assessment by judges on the subject is important to avoid the construction of material with superficial or briefly exposed content, compromising the effectiveness of the technology^(27)^. The judges, who validated the video, had direct experience with vaccination and, therefore, were able to contribute with relevant suggestions for improving the video. It is extremely important to validate the script for the educational video, since validity provides a sharper look from people with experience in the proposed topic, increasing the reach of the material being produced^(28)^.

Health education videos have been used as an alternative for teaching that provides a meaningful experience for participants^(29-30)^. It is an accessible way of bringing information in an interactive and dynamic way, being able to educate, communicate and inspire the public as a non-linear form of teaching, different from traditional forms, being able to sensitize and catch viewers’ attention^(21)^.

Thus, it is considered that the almost nullity of disagreements between experts and the perception of good understanding and scope of the content of the video produced may be related to the methodological rigor covered in this study. The judges agreed with the forms of prevention present in script/storyboard, since they follow the vaccination process proposed by the Brazilian PNI, suggesting forms of prevention that can be carried out in vaccination practice, in addition to considering a clear and objective language.

As for the content addressed in this video, it is understood that it is a difficult topic to discuss, due to the relationship with the idea of punishment and blaming professionals. IE are a reality faced by health systems and nursing, and measures for prevention, such as the adoption of protocols and checklists in vaccination rooms, investments in information technology, permanent education of professionals and detection of risk factors for the occurrence of errors, are fundamental^(9)^.

IE as well as other errors are caused by the interaction of several factors related to the professional, the environment and the institution/organization^(31)^. The contents addressed in the video point to several aspects of prevention, such as the correct administration of vaccines by professionals, such as careful reading of labels, double-checking the vaccine (professional and user) and avoiding distractions and interruptions. These actions are opportunities to transform individual attitudes, in order to be protective in their uniqueness, and collective attitudes, for the entire team’s safety^(28)^.

Another point addressed in the video was the importance of the institution providing enough products, inputs and human resources, as the responsibility for developing error prevention strategies is not exclusive to health professionals. The improvement of working conditions, such as a sufficient number of workers and suitable structure, guarantees quality patient care and professional safety^(32)^.

To err is human, hence the importance of error reporting. IE reporting is an important trigger to expose problems and risks that must be analyzed for the implementation of systematic and strategic changes to improve safety and quality of care^(33-34)^. Studies point to the incompleteness of reporting forms^(33,35-36)^, which causes a loss in case assessment, in addition to possible underreporting^(33)^.

The elaborated video reinforces the importance of error reporting, which can be the first attitude, in the sense of promoting users’ and professionals’ safety. It is important to encourage health professionals to reflect on their work in order to carry out actions that value their safety^(32)^.

### Study limitations

As a study limitation, the video validity with the target audience of only one municipality is pointed out, and there can be no generalization.

### Contributions to nursing, health or public policies

Vaccination activities in the Brazilian public service are still specific to nursing professionals. The educational video construction and validity about IE prevention emerges as a technological resource that can be incorporated into nurses’ educational strategies, contributing to safety in the vaccination room.

## CONCLUSIONS

The educational video for IE prevention was created and considered valid by judges and professionals working in vaccination rooms.

The forms of prevention described in the video addressed successes in administering vaccines, such as carefully reading labels and double-checking the vaccine (professional and user). It was pointed out the risk of distractions and interruptions for error occurrence and the need for the health institution to offer products, inputs and sufficient human resources, in addition to reinforcing the importance of reporting for the identification of causes and improvements in quality of care in the vaccination room. Video is a technological resource that facilitates the teaching-learning process that can be used to train professionals working in vaccination rooms.
